# Supplementary material for: Experimental Schistosoma japonicum-induced pulmonary hypertension
Source: PLoS Negl Trop Dis. 2022 Apr 13;16(4):e0010343. doi: 10.1371/journal.pntd.0010343 (PMC9037943; doi:10.1371/journal.pntd.0010343)
Supplement: Attachment — Submitted filename: sj_rebuttal_2022_03_10a.pdf [file pntd.0010343.s002.pdf]

PNTD-D-21-01490R1 Experimental Schistosoma japonicum-induced pulmonary hypertension

Point-by-Point Response to the Reviewers (Rebuttal text is in Bold)

Methods -Are the objectives of the study clearly articulated with a clear testable hypothesis stated? -Is the study design appropriate to address the stated objectives? -Is the population clearly described and appropriate for the hypothesis being tested? -Is the sample size sufficient to ensure adequate power to address the hypothesis being tested? -Were correct statistical analysis used to support conclusions? -Are there concerns about ethical or regulatory requirements being met?

Reviewer #1: The authors presented a very interesting study comparing the potential of two different species of Schistosoma in inducing pulmonar hypertension in mice. The study is of particular relevance since provide potential explanations to the difference in epidemiology of pulmonary hypertension associated to each one of the species (japonicum and mansoni).

The methodology is adequate, considering the knowledge about SchPH models so far

Rebuttal: Thank you for the positive comments.

Reviewer #2: The objectives are clearly articulated and the hypotheses were tested accordingly.

Rebuttal: Thank you for the positive comments.

Results -Does the analysis presented match the analysis plan?-Are the results clearly and completely presented?-Are the figures (Tables, Images) of sufficient quality for clarity?

Reviewer #1: The results are in accordance with the proposed methodology. Maybe the legends of the figures would benefit from a review to let the figure self-explanatory (some of the acronyms used in the figures are missing in the legend)

Rebuttal: Thank you for the comments, which we appreciate: the figure legends have been revised to be more self-explanatory.

Reviewer #2: The figures are well presented.

The analysis follows the plan stated.

However, the description of methods (with use of references) within the RESULTS section needs to be reviewed.

Rebuttal: Thank you for the comment. The results section text has been revised to remove descriptions of methods, and associated references; where appropriate the methods section has been revised to incorporate this text and citations.

Conclusions -Are the conclusions supported by the data presented?-Are the limitations of analysis clearly described?-Do the authors discuss how these data can be helpful to advance our understanding of the topic under study?-Is public health relevance addressed?

Reviewer #1: Conclusions are well based by the results and bring up the perspective of the findings

Rebuttal: Thank you for the positive comments.

Reviewer #2: Yes the conclusions arise from the results.

The limitations are described correctly.

Rebuttal: Thank you for the positive comments.

Editorial and Data Presentation Modifications? Use this section for editorial suggestions as well as relatively minor modifications of existing data that would enhance clarity. If the only modifications needed are minor and/or editorial, you may wish to recommend “Minor Revision” or “Accept”.

Reviewer #1: Some points would benefit of clarification / exploration if the data are available.

- the authors explored the quantity of eggs per tissue and also the thickness of the granuloma peri-egg. I wonder if the authors have quantified the number of granulomas per slide - as another form to support that S. japonicum might have a different immunogenicity

Rebuttal: Thank you for the comment. We have performed the requested analysis by quantifying the number of granulomas per slide, divided by the area (mm2) of tissue analyzed, and have included this as revised Figure 4B (and associated text in the Results section). The result was no significant difference between the 4 groups (Sm/Sm; Sj/Sj; Sj/Sm; Sm/Sj). Please note that per stereological principles, the number of observed 3-dimensional objects captured in a representative 2-dimensional plane is the mathematical product of the number of objects present and the average size of the objects (eg, see Chapter 5 in: Howard and Reed. Unbiased Stereology 2nd Edition. 2005, Garland Science/BIOS Scientific Publishers, NY, NY). For the specific example of comparing Sm/Sm to Sj/Sj, we interpret the result of no difference in granuloma number visualized as arising from the mathematical

product of a non-significant trend towards more eggs (Figure 4A) and a significant decrease in granuloma volume (Figure 3A), respectively, in the Sj/Sj group as compared to the Sm/Sm group.

- in sensitized mice, there was no difference in IL-4. Although the authors raised the potential limitation caused by the number of animals in each group, another potential reason could be a more limited role of IL4 it self in the inflammatory cascade triggered by schistosoma. The discussion would benefit of a paragraph trying to better explain the role of each one of the ILs (4 and 13) and the plausibility of the different roles according to the different species

Rebuttal: Thank you for the comment, indeed there are important differences between IL-4 and IL-13 which we had not directly addressed. The potential discrete roles of IL-4 and IL-13 have been discussed in a new paragraph in the discussion section.

- The difference in sensitized and non sensitized was an elegant way to reinforce the concept that this model is immunologically driven. It would be very nice if the authors have any data showing translation of these immunological phenomena to endothelial function. This could support potential differences between patients continuously exposed to schistosoma as compared to those already outside endemic regions

Rebuttal. Thank you for the comment, which raises an interesting link between host immunity and phenotype of non-immune cells, such as endothelial cells. We have not previously assessed endothelial cell phenotype in *Schistosoma*-PH. Others have investigated endothelial cell phenotype in *Schistosoma* more broadly (eg Oliveira et al. PLOS One 2011; ref #36 in the revised manuscript), and it makes sense that endothelial cells may be critical mediators of different pathologic phenotypes as these cells directly contact the embolized eggs when they lodge in the pulmonary vasculature. A section in the discussion section has been added to raise this concept, and we will investigate this possibility in future studies.

minor comment - in the author summary, japonicum is mentioned twice in the same sentence, where mansoni should be the comparator.

Rebuttal: Thank you catching this typo, which has been corrected.

Reviewer #2: Remove text for methods and remove discussion from the results section.

Rebuttal: Thank you for the comment, which we appreciate. Text describing methods or discussion of results has been moved from the results section to more appropriate places in the manuscript.

Summary and General Comments. Use this section to provide overall comments, discuss strengths/weaknesses of the study, novelty, significance, general execution and scholarship. You may also include additional comments for the author, including concerns about dual publication, research ethics, or publication ethics. If requesting major revision, please articulate the new experiments that are needed.

Reviewer #1: See above

Rebuttal: Thank you for the comment, which was addressed above.

Reviewer #2: This is a relevant topic and the authors should be congratulated for undertaking the study.

Rebuttal: Thank you for the positive comments.
